# MAP Kinase Regulation of the *Candida albicans* Pheromone Pathway

**DOI:** 10.1128/mSphere.00598-18

**Published:** 2019-02-20

**Authors:** Golnaz Rastghalam, Raha Parvizi Omran, Masoumeh Alizadeh, Debrah Fulton, Jaideep Mallick, Malcolm Whiteway

**Affiliations:** a Department of Biology, Concordia University, Montreal, Quebec, Canada; Carnegie Mellon University

**Keywords:** *Candida albicans*, GFP fusions, MAP kinase phosphatases, MAP kinases, pheromone pathway

## Abstract

We investigated the relationships of the Cek1 and Cek2 mitogen-activated protein (MAP) kinases and the putative MAP kinase phosphatase Cpp1 in the mating process of Candida albicans. Mutants of the *CPP1* gene are hyperresponsive to pheromone, generating large halos, high levels of projections, and an increase in pheromone-responsive gene expression. Mating-type-homozygous opaque cells that lack both kinases are sterile, consistent with previous observations, although several lines of evidence show that the two kinases do not simply provide redundant functions in the mating process. Loss of *CEK1* reduces mating significantly, to about 0.3% of wild-type strains, and also reduces projection formation and pheromone-mediated gene expression. In contrast, loss of *CEK2* has less of an effect, reducing mating to approximately one-third that of the wild-type strain and moderately reducing projection formation but having little influence on the induction of gene expression. However, loss of Cek2 function reduces adaptation to pheromone-mediated arrest. The mutation enhances pheromone response halos to a level similar to that of *cpp1* mutants, although the *cpp1* mutants are considerably more mating defective than the *cek2* mutant. The double *cek2 cpp1* mutant shows enhanced responsiveness relative to either single mutant in terms of gene expression and halo formation, suggesting the kinase and phosphatase roles in the adaptation process are independent. Analysis of protein phosphorylation shows that Cek1 undergoes pheromone-mediated phosphorylation of the activation loop, and this phosphorylation is enhanced in cells lacking either the Cpp1 phosphatase or the Cek2 kinase. In addition, Cek1-GFP shows enhanced nuclear localization in response to pheromone treatment. In contrast, Cek2 shows no evidence for pheromone-mediated phosphorylation or pheromone-mediated nuclear localization. Intriguingly, however, deletion of *CPP1* enhances both the phosphorylation state and the nuclear localization of Cek2-GFP. Overall, these results identify a complex interaction among the MAP kinases and MAP kinase phosphatase that function in the C. albicans mating pathway.

**IMPORTANCE** MAP kinases and their regulators are critical components of eukaryotic signaling pathways implicated in normal cell behavior as well as abnormal behaviors linked to diseases such as cancer. The mating pathway of the yeast Saccharomyces cerevisiae was central in establishing the MAP kinase paradigm. Here we investigate the mating pathway in a different ascomycete, the fungal pathogen C. albicans. In this dimorphic fungus MAP kinases are also implicated in the mating response, with two MAP kinases apparently playing redundant roles in the mating process. This work establishes that while some level of mating can occur in the presence of a single kinase, the Cek1 kinase is most important for mating, while the Cek2 kinase is involved in adaptation to signaling. While both kinases appear to be themselves regulated by dephosphorylation through the action of the Cpp1 phosphatase, this process appears important for mating only in the case of Cek1.

## Abstract (precis)

MAP kinases and their regulators are critical components of eukaryotic signaling pathways implicated in normal cell behavior as well as abnormal behaviors linked to diseases such as cancer. The mating pathway of the yeast Saccharomyces cerevisiae was central in establishing the MAP kinase paradigm. Here we investigate the mating pathway in a different ascomycete, the fungal pathogen C. albicans. In this dimorphic fungus MAP kinases are also implicated in the mating response, with two MAP kinases apparently playing redundant roles in the mating process. This work establishes that while some level of mating can occur in the presence of a single kinase, the Cek1 kinase is most important for mating, while the Cek2 kinase is involved in adaptation to signaling. While both kinases appear to be themselves regulated by dephosphorylation through the action of the Cpp1 phosphatase, this process appears important for mating only in the case of Cek1.

## INTRODUCTION

The mating systems of ascomycete yeasts are typically simple, with two sexes, classically designated **a** and α. These opposite sexes communicate by means of small diffusible molecules known as pheromones, generally a lipopeptide pheromone in the case of the **a** mating type, and a simple peptide in the case of the α mating type (1). These pheromones are detected by cell type receptors on the surface of a potential partner, and their presence is communicated to the response machinery within the cell by means of a heterotrimeric G protein complex that, when activated by the pheromone-bound receptor, serves in turn to activate a downstream mitogen-activated protein (MAP) kinase cascade module (2–4). Although specific details of the relative roles of the proteins in these modules may differ among species, the general structures of the pathways appear quite well conserved.

In both the pre-genome-duplication yeasts like Candida albicans and the post-genome-duplication yeasts like Saccharomyces cerevisiae, the MAP kinase (MAPK) modules associated with the mating pathway contain a pair of highly similar protein kinases. This suggests that the duplication of the kinases preceded the whole-genome duplication (WGD; actually a fusion of two very related, but distinct strains) (5) that led to the S. cerevisiae lineage and that subsequent to the WGD the genome reverted to only two kinases. Intriguingly, the timing of the MAP kinase duplication is correlated with the appearance of an Ste5 mating pathway scaffold ortholog (6).

Although initial studies in S. cerevisiae suggested the two MAP kinases, known in yeast as Fus3 and Kss1, were redundant for mating (7), further investigations showed that the molecular roles of these proteins were distinct (8). In particular, careful studies in S. cerevisiae showed that activation of the Fus3 kinase by the upstream MEK Ste7 required interaction with the Ste5 scaffold; this scaffold interaction was not required for activation of Kss1 (9). The requirement for Ste5 interaction in the activation of Fus3 linked Fus3 specifically to the mating pathway; the apparent redundancy of the kinases arose because Kss1, which can be activated directly by Ste7 without Ste5 intervention, could replace enough of Fus3 function in the absence of Fus3 to allow a significant level of mating (7). This replacement of Fus3 by Kss1 was quite effective in a Fus3 deletion strain but was much less effective in cells that had a structurally intact, but kinase-domain-inactivated, Fus3 protein (10).

Because the von Willebrand factor domain of the Ste5 scaffold protein required for the activation of Fus3 in S. cerevisiae is missing in the Cst5 scaffold protein in C. albicans (6), it is likely that the relationship between the two related MAP kinases (designated Cek1 and Cek2) in the C. albicans mating pathway is distinct from that established for Fus3 and Kss1 in S. cerevisiae. Initial studies suggested that the Cek1 and Cek2 kinases were redundant for mating in C. albicans (11). More recent studies have established quantitative differences in the importance of each kinase in the mating pathway and confirmed that the kinases are also redundant for mating when the cells were in the opaque state (12). In these studies, the Cek1 kinase, which was isolated in a manner similar to Kss1 (13), was found to be quantitatively most important for mating, while Cek2 had a quantitatively less important role in the mating process (12).

Previous work had shown a role for the Cek1 kinase in hyphal growth on Spider medium and showed that the candidate MAP kinase phosphatase Cpp1 acted antagonistically to the kinase (14). A recent study has also implicated Cek1 as the target of Cpp1 in stimulating hyperfilamentation and the switching of *MTL* homozygous cells to the opaque phenotype during growth on glucosamine medium and has also addressed the biochemical relationship between Cpp1 and Cek1 (15). Here we have investigated whether Cpp1 also acts antagonistically to Cek1 and/or Cek2 in the mating response pathway. We find that loss of Cpp1 activates the pheromone response pathway in a Cek1-dependent manner, supporting its role as a MAP kinase phosphatase in the mating pathway. Surprisingly, activation of the pheromone response pathway resulting from loss of Cpp1 is not suppressed by deletion of Cek2; in fact, the double mutant strain is more highly responsive than the *cpp1* mutant alone. This shows that the two MAP kinases, although ultimately redundantly required for mating, play clearly distinct roles in the C. albicans mating process.

## RESULTS

### Cpp1 is a negative regulator of the mating pathway.

**FIG 1:**
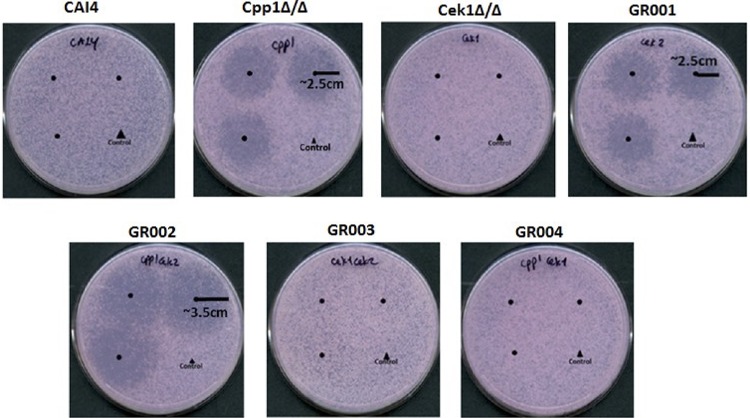
Halo assays with *C. albicans* strains. Halo assays were performed by plating a lawn of opaque **a** cells on SC-glucose plates and spotting α-pheromone (1 μg) and a control (50% methanol) onto each plate. Pheromone was spotted on the center of each of three circles drawn on the plates, and the 50% methanol control was spotted on the fourth spot. Plates were incubated at room temperature for 2 days for the lawn of cells to grow and then were photographed. Halo activities of opaque cells from mutants were compared to wild-type strains. Cpp1Δ/Δ, GR001, and GR002 are hyperresponsive to pheromone treatment as they generate large halos compared to the wild-type strain, which generates almost imperceptible halos in a standard halo assay test. The size of the halos was measured over the plates.

**FIG 2:**
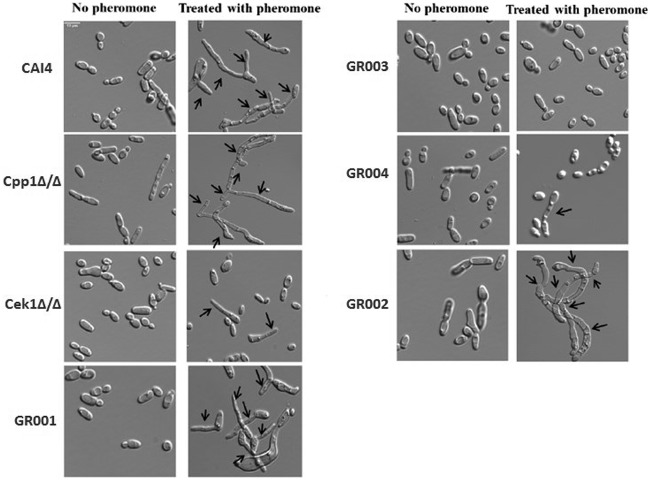
Pheromone response assay. Opaque cells were treated with α-pheromone for 3 hours, 6 hours, and 24 hours. This image shows only the results for 24 h of treatment. Cells were observed under a Leica DM6000 microscope. α-pheromone does not induce conjugation tube formation (shmoos) in opaque cells of the Cek1Δ/Δ and GR004 mutants after 3 h and 6 h of treatment, and less than 0.1% of cells showed tube formation after 24 h of treatment. GR003 never induced conjugation tube formation. Arrows designate conjugation structures.

**FIG 3:**
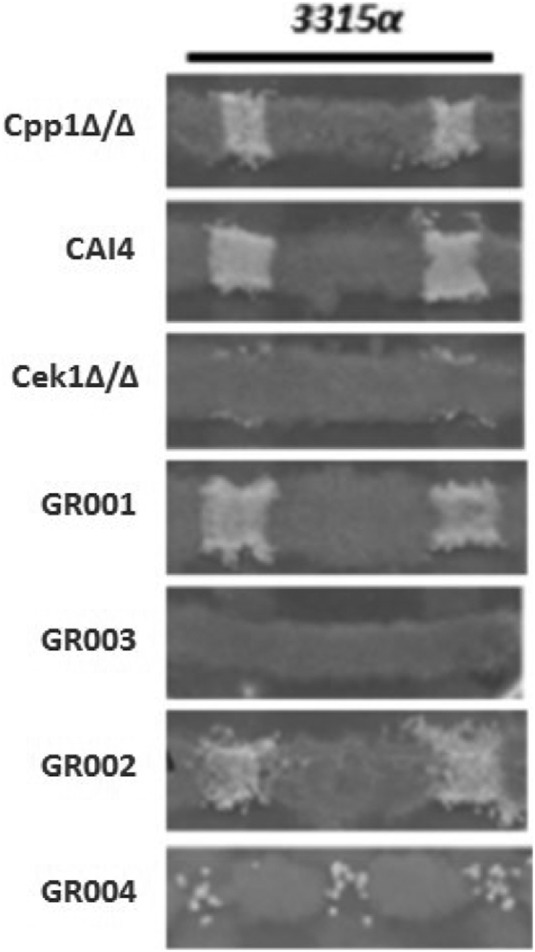
SC-glucose medium mating assay. Strain 3315α (*trp1/trp1 lys2/lys2*) in the opaque state was used as the mating type tester for cross print mating assays with WT and mutant *MTL***a** strains. This tester was crossed with mutant strains on SC-glucose agar medium at 24°C for 2 days and then replicated on SC-glucose selection medium (Trp^−^ Lys^−^ Ura^−^) at 30°C for 3 days to detect prototrophic mating products.

To investigate whether the Cpp1 phosphatase functions in the pheromone response pathway, we constructed a mating-competent *cpp1* null mutant strain by identifying an *MTL* homozygous version of strain CP29-1-7L4 (14) by sorbose selection (16, 17). Table 1 gives a list of strains. We confirmed the *cpp1* mutation and the *MTL* phenotype by PCR. We tested this mutant for modifications in response to pheromone and ability to mate. *cpp1*Δ/Δ mutants are hyperresponsive to pheromone treatment as they generate large distinct halos compared to the wild-type (WT) strain CAI4, which generates almost imperceptible halos in a standard halo assay test (Fig. 1). Direct microscopic assessment of projection formation showed that the *cpp1*Δ/Δ mutant formed more frequent and extensive projections than the control strain (CAI4) (Fig. 2 and Table 2) and, even in the absence of pheromone, showed frequent elongated cells. Furthermore, RNA-seq analysis of pheromone-treated *cpp1*Δ/Δ mutant strains showed that the deletion of *CPP1* makes C. albicans more responsive to pheromone treatment, inducing a set of selected pheromone-responsive genes (with the exception of the missing *CPP1* gene) to a level generally about 3-fold greater than the wild-type strain (Table 3). However, even though the cells were more pheromone responsive, qualitative (Fig. 3) and quantitative (Table 4) mating experiments showed that loss of *cpp1* actually reduces mating to about 2% that of the wild-type strains.

**TABLE 1:** Strains used in this study

Strain	Parent	Mating type	Description	Genotype	Source or reference
CAI4 *MTL***a**	CAI-4	**a**/**a**	*ura3*::*imm434/ura3*::*imm434*	Wild type	Doreen Harcus
CP29-1-7L4	CAI-4	**a**/α	*ura3/ura3 cpp1*::*hisG/cpp1*::*hisG*		26
Cpp1Δ/Δ	CP29-1-7L4	**a**/**a**	*ura3/ura3 cpp1*::*hisG/cpp1*::*hisG*	*cpp1*Δ/Δ	This study
CK43B-16L		**a**/α	*ura3/ura3 cek1*::*hisG/cek1*::*hisG*		14
Cek1Δ/Δ	CK43B-16L	**a**/**a**	*ura3/ura3 cek1*::*hisG/cek1*::*hisG*	*cek1*Δ/Δ	This study
GR001	CAI4 *MTL***a**	**a**/**a**	*ura3/ura3 cek2*::*TAA* (32nd and 37th amino acids)/*cek2*::*TAA* (32nd and 37th amino acids)	Cek2-stop/stop	This study
GR002	Cpp1Δ/Δ	**a**/**a**	*ura3/ura3 cpp1/cpp1*; *cek2*::*TAA* (32nd and 37th amino acids)/*cek2*::*TAA* (32nd and 37th amino acids)	Cpp1Δ/Δ Cek2-stop/stop	This study
GR003	Cek1Δ/Δ	**a**/**a**	*ura3/ura3 cek1/cek1*; *cek2*::*TAA* (32nd and 37th amino acids)/*cek2*::*TAA* (32nd and 37th amino acids)	Cek1Δ/Δ Cek2-stop/stop	This study
GR004	Cpp1Δ/Δ	**a**/**a**	*ura3/ura3 cpp1/cpp1*; *cek1*::*TAA* (19th and 25th amino acids)/*cek1*::*TAA* (19th and 25th amino acids)	Cpp1Δ/Δ Cek1-stop/stop	This study
Cpp1Δ/Δ (Cek1-GFP)	Cpp1Δ/Δ	**a**/**a**		Cpp1Δ/Δ (Cek1-GFP)	This study
CAI4, Cek1-GFP	CAI-4	**a**/**a**		Cek1-GFP	This study
Cpp1Δ/Δ (Cek2-GFP)	Cpp1Δ/Δ	**a**/**a**		Cpp1Δ/Δ (Cek2-GFP)	This study
CAI4 (Cek2-GFP)	CAI-4	**a**/**a**		Cek2-GFP	This study
3315	A505	α/α	*trp1/trp1*; *lys2/lys2*		P. T. Magee
SN148	RM1000#2	**a**/α	*arg4/arg4*; *leu2/leu2*; *his1/his1*; *ura3 imm434/ura3 imm434*; *iro1* *imm434/iro1 imm434*		Suzanne Noble/Sandy Johnson

**TABLE 2:** Percentage of projection formation for different strains^*a*^

Strain	Duration of pheromone treatment (h)	No. of cells showing morphological projection	% of projection formation
CAI4	3	57	50
	6	81	85
	24	100	100
Cpp1Δ/Δ mutant	3	91	94
	6	98.5	100
	24	100	100
Cek1Δ/Δ mutant	3	2	1.5
	6	2.1	4
	24	7.5	10
GR001	3	21	28
	6	56.5	58
	24	90.5	92
GR002	3	95.5	98
	6	99	100
	24	100	100
GR003	3	0.00	0.00
	6	0.00	0.00
	24	0.00	0.00
GR004	3	2.4	5
	6	4	8
	24	9.5	12

a The percentage of projection formation was computed by counting the number of cells showing morphological projections after treating the cells with pheromone for 3, 6, and 24 h divided by the total number of cells counted (∼150 cells for each strain) and multiplying by 100. All cells were of the opaque type.

**TABLE 3:** RNA sequence analysis^*a*^

Gene	ORF	Induction of gene in strain:						
		CAI4 (18)^*b*^	Cpp1Δ/Δ mutant (19)	Cek1Δ/Δ mutant (0)	GR001 (17)	GR003 (0)	GR002 (20)	GR004 (2)^*c*^
PRM1	19.8286	13	25	0	10	0.8	182	0
FIG1	19.138	45	154	0	17	0	659	82
SST2	19.4222	7	19	0.6	6	0.5	116	0.9
RBT1	19.1327	13	72	0.7	7	0	93	5
HWP1	19.1321	8	40	0	9	0.1	37	0.2
CEK2	19.46	6	10	0.9	5.2	0.5	19	1.7
STE2	19.696	9	18	0.3	10	0.3	52	1.2
RBT4	19.6202	15	92	0.6	18	0.4	120	2.3
KAR4	19.3736	9	26	0.6	6	0.9	93	6
RAM1	19.5046	13	32	0.6	19	0.6	108	1.8
CEK1	19.2886	4	11	0.6	8	0.2	34	0
PCL1	19.2649	7	38	0.2	7	1.5	91	8
CPP1	19.4866	2.5	0.84	1.4	1.8	0.9	2	0.2
FAV1	19.3801	12	56	0.8	30	0.6	127	5
FAV2	19.112	25	73	0.7	14	0	65	4.5
FAV3	19.1914	3	12	0.9	1.4	0.6	60	2
ASG7	19.552	19	11	0	8	0	61	0.7
FGR23	19.1616	26	50	2.6	25	0.9	189	6
C2_07220	19.2278	1.4	25.6	1.3	1.8	0.8	59	16
DAG7	19.4688	4	12	0.7	4	0.5	24	1.7
POL	19.2219	18	20	1.2	10	0.9	54	2.9

a Twenty-one pheromone-induced genes from the literature (18) were selected as the reference set for assessing the pheromone response of various strains. RNA-seq results were normalized against the WT CAI4 strain not treated with pheromone. Selected genes were defined and were counted as upregulated if they were located in the top 200 genes ranked by fold increase over control for the strain.

b Numbers in parentheses after strain names are the numbers of upregulated genes out of 21 pheromone-induced selected genes.

c FIG1 and C2_07220 genes are induced in GR004.

**TABLE 4:** Quantitative mating assays^*a*^

Strain	Mating partner	Mating %	% of mating relative to WT (CAI4)
CAI4	3315α	23.5 ± 1.5	100
Cpp1Δ/Δ mutant	3315α	0.33 ± 0.03	1.4
Cek1Δ/Δ mutant	3315α	0.07 ± 0.02	0.3
GR001	3315α	8.5 ± 0.5	36
GR002	3315α	0.37 ± 0.02	1.6
GR003	3315α	0	0
GR004	3315α	0.09 ± 0.05	0.4

a Tester strains and experimental strains were precultured in SC-glucose liquid medium for 24 h and then mixed in fresh liquid SC-glucose medium at a concentration of 1 × 10^7^ cells/ml for all strains. Mixed cells were incubated at RT for 48 h and then plated onto selection medium to detect auxotrophic mating products. The mating frequency is calculated as described in Materials and Methods. Opaque cells of the mutant GR003 do not mate with opaque cells of the opposite mating type 3315α strain, whereas Cek1Δ*/*Δ and GR002 mutant strains mate but at reduced frequency. Percentage of reduction in mating was computed by subtracting the percentage of mating of different mutant strains from the percentage of the mating of the wild-type (CAI4) strain divided by the percentage of the mating of the wild-type (CAI4) strain, and the fraction was multiplied by 100.

### Does the hyperresponsiveness of the *cpp1*Δ/Δ null mutant require MAP kinase function?

**(i) Cek1.** We created a *cek1* mutant (strain Cek1Δ/Δ) and a double mutant of *cek1* and *cpp1* (strain GR004) to assess whether the Cek1 kinase is needed for the hyperactivity observed for the *cpp1*Δ/Δ null mutant during pheromone response. As previously observed (11, 12), we found the Cek1 kinase to be important, but not absolutely essential, for C. albicans pheromone response and mating. Quantitative mating assays show that strain Cek1Δ/Δ reduces mating to about 0.3% that of the wild-type strain CAI4 (Table 4). This level of mating allows strain Cek1Δ/Δ to form detectable prototrophic mating products in cross print mating assays with the tester *MTL*α/α strain 3315 (Fig. 3). However, the Cek1Δ/Δ strain shows greatly reduced projection formation in response to pheromone treatment (Fig. 2 and Table 2), and it does not undergo efficient pheromone-mediated cell cycle arrest and thus does not make halos in a standard halo assay (Fig. 1). As well, when assessed for pheromone-induced gene expression, the Cek1Δ/Δ strain fails to induce any of a set of genes chosen from the literature (18) to represent the pheromone-induced regulon (Table 3).

Strain GR004 was generated by inactivating the *CEK1* gene in the Cpp1Δ/Δ strain using CRISPR as described in Materials and Methods. Loss of the Cek1 function eliminated the hyperresponsiveness of the *cpp1* mutant. The double mutant showed no halos (Fig. 1) and had reduced projection formation relative to the wild type in response to pheromone (Fig. 2 and Table 2). Strain GR004 reduces mating to about 0.4% that of the wild-type strains, similar to that of the *cek1* mutant alone (Table 4). As also shown in Fig. 3, strain GR004 forms infrequent prototrophic mating products in cross print mating assays with the tester *MTL*α/α strain. Finally, the double mutant showed poor induction of the pheromone-induced regulon, with most of the regulon genes showing weak or no induction in the presence of pheromone (Table 3). Thus, the Cek1 kinase is essential for the hyper-pheromone-responsiveness shown by the Cpp1Δ/Δ strain, while removal of *cpp1* does not dramatically enhance the mating response of the *cek1* mutant.

**(ii) Cek2.** We similarly investigated the role of the Cek2 kinase in the pheromone hypersensitivity of the *cpp1* mutant. Previous work (12) had suggested that the Cek2 MAP kinase was a minor player in pheromone response. However, recent studies on artificially activating mating in a white cell background found a major role for Cek2 in the process (19). In the SN148 strain background, loss of Cek2 (strain GR001) generated a minor effect on overall mating, reducing mating efficiency to approximately 35% that of the wild-type strain (Table 4 and Fig. 3). Loss of *CEK2* also moderately reduced projection formation relative to the wild type (Fig. 2 and Table 2) but had little effect on induction of the pheromone regulon, with most of the regulon genes having induction levels similar to that observed in the wild-type strain (Table 3). Intriguingly, loss of Cek2 function considerably reduced adaptation to pheromone-mediated cell cycle arrest, resulting in the formation of large halos (Fig. 1); these halos were similar in size to those generated by loss of Cpp1.

We created a *cek2 cpp1* double mutant (strain GR002) to assess the relationship between this MAP kinase and the MAP kinase phosphatase. Cek2 was not needed for the enhanced responsiveness shown by the *cpp1* mutant; in fact, the *cpp1 cek2* double mutant showed further enhanced responsiveness relative to either single mutant. The double mutant generated large halos (Fig. 1), somewhat larger than those formed by either of the single mutants. The double mutant strain mated at about 2% the rate of the wild-type strain, similar to strain Cpp1Δ/Δ but lower than that of the *cek2* mutant alone (strain GR001) (Table 4 and Fig. 3). The double mutant also formed a high level of projections (Fig. 2 and Table 2) and showed a very strong induction of the pheromone regulon (Table 3). Thus, loss of the Cek2 MAP kinase does not reduce the enhanced pheromone response of the Cpp1Δ/Δ mutant strain; in fact, it further stimulates the response.

### MAP kinase function in mating.

Because we had found that loss of *cek2* in the SN148 background enhanced aspects of pheromone response while reducing mating, we examined the *cek1 cek2* double mutant (strain GR003) for mating and pheromone response. As found previously (11, 12) the *cek1 cek2* double mutant was completely nonresponsive and nonmating. As shown in Fig. 3, this strain fails to show formation of prototrophic mating products in cross print mating assays with the tester *MTL*α/α 3315 strain, or to generate prototrophic cells in quantitative mating assays (Table 4). The cells also fail to make projections in response to a pheromone treatment (Fig. 2 and Table 2), and they do not undergo pheromone-mediated cell cycle arrest and make halos in a standard halo assay (Fig. 1). Finally, the double mutant was totally unable to induce the pheromone regulon (Table 3).

### Biochemical assessment of Cpp1 function.

**FIG 4:**
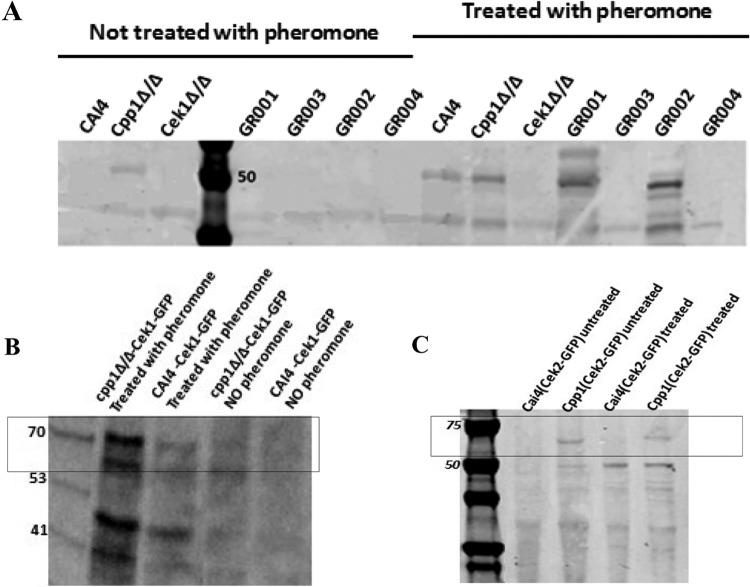
Activation loop phosphorylation. Protein extracts from untreated strains and strains treated with α-pheromone were probed with an anti-phosphorylated-activation-loop antibody (anti-P44/42 MAPK polyclonal antibodies [Cell Signaling catalog no. 9101]) that detects the phosphorylated form of the Cek1 and Cek2 MAP kinases. (A) In the untreated lanes, only the strain lacking Cpp1 shows any phosphorylated MAP kinase; this band may be derived from phospho-Cek1 or -Cek2 because it is missing in the *cpp1* mutant strains lacking either kinase. After pheromone treatment, the MAP kinase band shows enhanced phosphorylation in the WT strain; this appears to result from Cek1 because it is missing in all the *cek1* deletion strains and enhanced in the *cek2* deletion strain. This band is increased in the pheromone-treated Cpp1Δ/Δ strain and in the GR002 strains. To distinguish bands derived from Cek1 from those derived from Cek2, we examined the phosphorylation status of GFP fusions to the Cek1 and Cek2 kinases. (B) One allele of Cek1 is tagged with GFP in Cpp1Δ/Δ and CAI4 (WT) strains. Cek1-GFP activation site phosphorylation is enhanced by pheromone treatment and further enhanced by loss of the Cpp1 phosphatase. (C) Cek2 activation site phosphorylation is not detected in the untreated or the pheromone-treated WT strain but is enhanced equally in pheromone-treated or untreated cells lacking the Cpp1 phosphatase. (We loaded equivalent amounts of protein in each lane based on protein quantification by the Bradford assay.) Numbers in gels are molecular masses in kilodaltons.

We investigated whether Cpp1 influenced phosphorylation of Cek1 in response to pheromone signaling using an antibody against a phosphorylated MAP kinase activation loop. As shown in Fig. 4A and B, we detected enhanced phospho-Cek1 in strain Cpp1Δ/Δ compared to the wild-type strain (CAI4) in the absence of pheromone and an even stronger phospho-MAPK band in the pheromone-treated strain Cpp1Δ/Δ. Therefore, consistent with previous observations (15), the *CPP1* deletion resulted in Cek1 hyperphosphorylation, implicating Cpp1 as a regulator of the mating MAPK cascade through its action on Cek1. Intriguingly, the absence of Cek2 also enhances the phosphorylation of the MAPK Cek1 compared to the wild-type CAI4 (Fig. 4A). Finally, strain GR002 showed an increased phosphorylated form of the MAPK compared to the wild-type (WT) CAI4 and either the single mutant Cpp1Δ/Δ strain or GR001 (Fig. 4A). We do not detect any phospho-MAPK band in any of the *cek1Δ/Δ* strains. This suggests the activation loop phosphorylation antibody is not detecting a signal from Cek2 in pheromone-treated *cek1* strains.

### MAP kinase activation loop phosphorylation of Cek1-GFP and Cek2-GFP.

We directly investigated the phosphorylation status of Cek1 and Cek2 in response to pheromone treatment and to the presence of the Cpp1 phosphatase by separately tagging each kinase with GFP. Because the tagged kinase runs at a location distinct from the wild-type protein, we could observe the phosphorylation status of each kinase independently. As shown in Fig. 4B, the GFP-tagged Cek1 showed enhanced activation loop phosphorylation in response to pheromone treatment and in response to deletion of *CPP1*, similar to the situation previously identified for the untagged protein. Intriguingly, as shown in Fig. 4C, the GFP-tagged Cek2 showed no evidence for any activation loop phosphorylation in response to pheromone treatment and no detectable basal signal in the untreated wild-type strain. However, the loss of Cpp1 resulted in detectable activation loop phosphorylation; this signal was not enhanced by pheromone treatment (Fig. 4C).

### Cell biological assessment of Cpp1 function.

**FIG 5:**
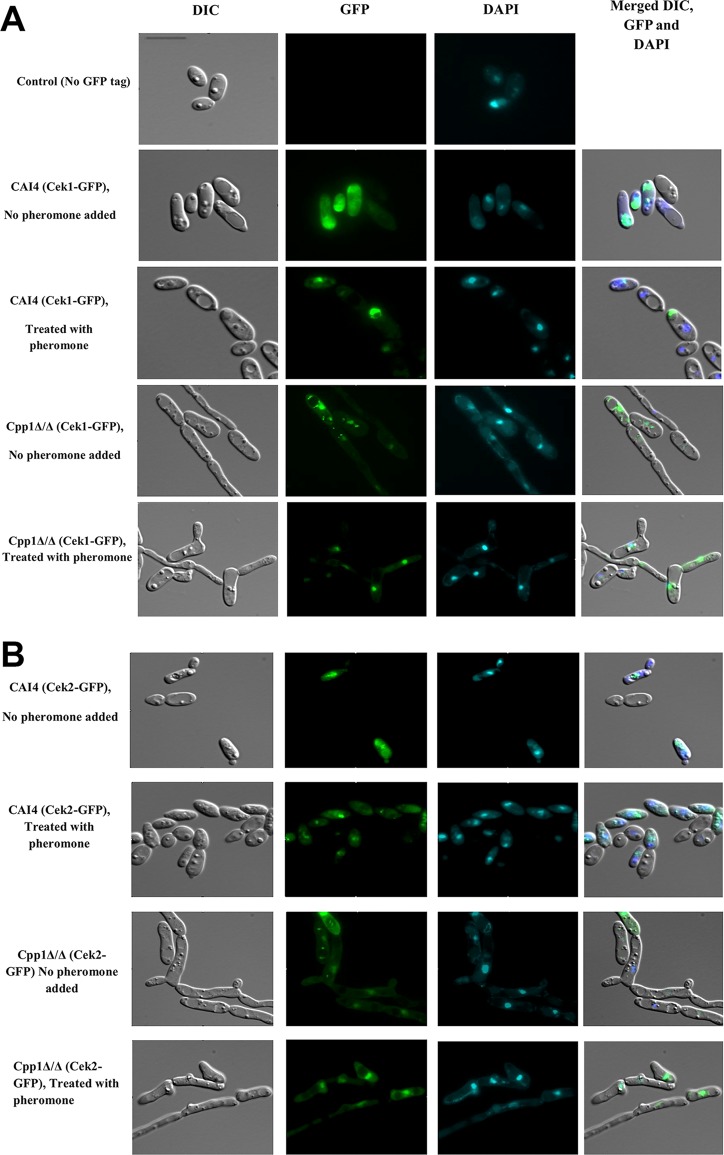
Localization of Cek1-GFP and Cek2-GFP before and after pheromone treatment. Cek1-GFP in both CAI4 and Cpp1Δ/Δ strains shows enhanced nuclear localization in response to pheromone treatment. In contrast, Cek2-GFP shows no evidence for pheromone-mediated nuclear localization. However, deletion of *CPP1* enhances the nuclear localization of Cek2-GFP in the Cpp1Δ/Δ strain compared to the CAI4 wild-type strain. Bar, 13 μm.

We used the GFP-tagged Cek1 and Cek2 proteins to localize the proteins in WT (CAI4) and Cpp1Δ/Δ strains either treated with pheromone or untreated. As shown in Fig. 5, untreated wild-type strains show the Cek1-GFP fusion protein distributed throughout the cell. This distribution appears less dispersed in the untreated strain Cpp1Δ/Δ, which shows enhanced nuclear localization of the fusion protein. Treatment of either the wild-type strain or the *cpp1Δ/Δ* strain with pheromone enhanced the cellular localization of the Cek1-GFP fusion protein to the nucleus, as confirmed by the colocalization of the GFP signal with the DAPI staining. Overall, enhanced activation loop phosphorylation, generated by either pheromone treatment, deletion of *CPP1*, or both, enhanced localization of Cek1-GFP to the nucleus.

Localization of the Cek2-GFP differs from that of Cek1-GFP. For wild type (CAI4), both untreated and treated cells show a clear intracellular concentration, but this is typically perinuclear rather than nuclear. However, in *cpp1* mutant strains, both treated and untreated cells show localization of the Cek2-GFP in the nucleus. Thus, both kinases show enhanced nuclear localization correlated with activation loop phosphorylation; in the case of Cek1, this results from either pheromone treatment or loss of the Cpp1 phosphatase, but in the case of Cek2, pheromone treatment does not appear to influence nuclear localization.

## DISCUSSION

There is a complex relationship among the Cek1 and Cek2 MAP kinases and the MAP kinase phosphatase Cpp1 functioning in the C. albicans mating response. Evidence provided in this study and in previous work (12) suggests that the Cek1 kinase is the major MAP kinase required for the pheromone response. Loss of this kinase greatly compromises all aspects of the mating response, reducing processes such as induction of pheromone-responsive genes, projection formation, and mating, far below the wild-type level. However, the loss of Cek1 function does not create complete sterility, as a residual level of morphological response and mating can be detected in *cek1Δ/Δ* strains that is dependent on the related MAP kinase Cek2. As previously noted, we find that deletion of both kinases completely blocks pheromone response and mating (11, 12).

This pattern of two kinases playing distinct roles but having overlapping functions that require both to be be deleted to generate sterility is similar to that seen for the MAP kinases Fus3 and Kss1 of the post-whole-genome duplication (WGD) yeast S. cerevisiae (5). The fact that Cek1/Cek2 paralogs are present in the pre-WGD yeast C. albicans establishes that the MAPK duplication preceded the WGD, occurring coincidentally with the appearance of an Ste5 equivalent (6). Therefore, during the WGD, duplication of the Cek1/Cek2 paralogs should have created 4 paralogous genes, a pair of *CEK1* orthologs, and a pair of Cek2 orthologs. To get to the 2-paralog pattern that exists in S. cerevisiae, two of these duplicated genes must be lost. Synteny, sequence similarity, and functional relatedness all are consistent with *FUS3* and *CEK1* being orthologs. The direct derivation of *KSS1* is less clear; however, it is likely that Fus3 and Kss1 are on the same phylogenetic branch with CaCek1, and both are diverged from CaCek2, suggesting that the Cek1 paralog of the pre-WGD yeast may have provided the lineage leading to both Fus3 and Kss1 in S. cerevisiae. In this context, it is interesting that the identification of CaCek1 (13) involved an S. cerevisiae screen similar to that which identified Kss1 (20), while Cek1 also appears to provide a Fus3-like major function in the C. albicans mating pathway (reference 12 and this paper).

The function of the Cek1 MAP kinase in the pheromone response appears regulated by the Cpp1 phosphatase. In response to pheromone treatment, the activation loop of Cek1 shows enhanced phosphorylation, and the level of phosphorylation in both pheromone-treated and untreated cells is enhanced by the loss of the Cpp1 phosphatase. Phenotypic analysis of the *cpp1Δ/Δ* strain shows that all aspects of pheromone response are modified in such strains—the cells show enhanced projection formation and pheromone-induced gene expression in response to pheromone, they show greatly increased pheromone-mediated cell cycle arrest measured by halo assays, and they show significantly reduced mating compared to nonmutant cells. As well, pheromone-treated cells or cells lacking Cpp1 show a greater concentration of Cek1 in the nucleus, consistent with activation loop phosphorylation being involved, directly or indirectly, in nuclear localization.

The Cpp1 phosphatase also appears to modulate Cek2 phosphorylation, but this process appears unrelated to mating signaling. In wild-type cells, the Cek2-GFP fusion construct shows clear intracellular concentration that appears next to the nucleus, and this localization is not influenced by pheromone treatment. However, deletion of the Cpp1 phosphatase results in the Cek2-GFP signal becoming more nuclear, suggesting that Cek2, like Cek1, shows enhanced nuclear localization correlated with enhanced activation loop phosphorylation. A role of Cpp1 in opposing MAP kinase activation loop phosphorylation is consistent with studies on the S. cerevisiae ortholog Msg5, which show the phosphatase can downregulate the mating signal and inhibit the nuclear localization of Fus3 (21).

We provide a speculative model for the roles of the MAP kinases and MAP kinase phosphatase in the C. albicans pheromone response. It appears that the major signaling pathway involves the receptor/G protein module activating the upstream elements leading to Hst7 phosphorylating Cek1 on the activation loop, and this in turn enhances nuclear localization of the kinase and activation of downstream events. This activation is opposed by the action of the Cpp1 phosphatase; in the absence of Cpp1, Cek1 activation is enhanced and sustained, leading to increases in pheromone-mediated gene induction, projection production, and halo formation. In the absence of Cek1, a low level of mating still occurs, and this mating is dependent on Cek2. However, overall Cek2 appears to play a minor role in the mating process: its most significant role in this process in wild-type cells appears to be to regulate reentry into the cell cycle after pheromone-mediated arrest. Overall, loss of Cek2 causes only a minor decrease in mating and in morphological changes in response to pheromone and has essentially no effect on pheromone-mediated gene induction. However, loss of Cek2 leads to a significant increase in pheromone-mediated cell cycle arrest as measured by halo assays. Because the frequency of projections in the mutant strain is not dramatically enhanced relative to WT cells, it is likely the larger halos result from an inability to adapt to pheromone treatment and to reenter the cell cycle. Proteins like Bar1, Sst2, or Ste50 (22, 23) are implicated in adaptation to the signal and important for reentry into the cell cycle after pheromone treatment in the related ascomycete S. cerevisiae; if the adaptation process in C. albicans involved a protein that needed Cek2-mediated phosphorylation for function, the absence of the kinase could generate enhanced halos by preventing proper function of the adaptation process. Because the loss of both Cpp1 and Cek2 leads to increased halos relative to either single mutant, the Cek2 function in adaptation does not appear to act through Cpp1.

The relationship among Cek1, Cek2, and Cpp1 is not limited to the mating pathway. Both kinases play roles in cell wall integrity control (24), and Cpp1 has been implicated in providing cross talk between the Hog1- and Cek1-controlled pathways implicated in morphological regulation (15). The increase in nuclear localization of both kinases in the absence of Cpp1 suggests activation loop phosphorylation plays a critical role in their regulation. In this context, the differential localization of the Cek1 and Cek2 kinases in the absence of stimulation is intriguing. Further work will be necessary to sort out the details of MAP kinase phosphorylation/dephosphorylation and cellular function, with the ultimate goal of understanding how a single MAP kinase with roles in different pathways is properly directed and regulated and how closely related kinases are connected to unique regulatory events.

## MATERIALS AND METHODS

### Strains, media, and culture conditions.

Strains used in this study are listed in Table 1. The C. albicans strains used for these experiments were all derivatives of CAI4 (*ura3*::*imm434*/*ura3*::*imm434*) (25). The *cek1*Δ/Δ **a**/α (CK43B-16L) (14) and *cpp1*Δ/Δ **a**/α (CP29-1-7L4) (26) strains are as described previously. Equivalent **a/a** strains were derived from the parent strains by sorbose selection (16).

*CEK2* was disrupted in the CAI4 **a/a**, Cpp1Δ/Δ **a/a**, and Cek1Δ/Δ **a/a** parent strains using a C. albicans CRISPR-Cas9 system (27).

*CEK1* was disrupted in the parental Cpp1Δ/Δ **a/a** strain using the same method (27). *cek1* and *cek2* were targeted using the oligonucleotides listed in Table S1 in the supplemental material.

10.1128/mSphere.00598-18.1 TABLE S1 List of oligonucleotides used in the study. Download Table S1, DOCX file, 0.02 MB. Copyright © 2019 Rastghalam et al. 2019 Rastghalam et al. This content is distributed under the terms of the Creative Commons Attribution 4.0 International license.

Cek1 and Cek2 were separately tagged with GFP in CAI4 and Cpp1Δ/Δ strains. These GFP fusion strains were created to investigate protein localization in response to pheromone by tracking of the GFP signal using a Leica DM6000 microscope.

Strains were plated on synthetic complete medium supplemented with 2% glucose or 1.25% GlcNAc and 100 µg/ml uridine at 25°C and grown in SC-liquid medium with glucose or GlcNAc at room temperature prior to assays. For opaque colony identification, Phloxine B (5 μg) (28) was added to solid SC glucose (2%) or GlcNAc (1.25%) medium.

### CRISPR-Cas9.

We used the “solo system” strategy for Candida albicans CRISPR-Cas9 (27). The pV1093 plasmid with *CAS9* and both ampicillin and nourseothricin as markers was used. Benchling was used to design the guide RNA selecting optimal PAM sites for the desired construct (27). The sgRNA (20 nucleotides) has extended sequences on both 5′ and 3′ ends to allow cloning into the BsmBI site of plasmid PV1093: forward, 5′-ATTTGX20g-3′, and reverse, 5′-AAAACX20c-3′. The repair DNA was also designed through Benchling from homologous sequences flanking the sgRNA target sequence. The repair DNA is modified to include two in-frame stop codons (TGA used in this mutant), a disrupted PAM region (NGG), and introduction of a restriction enzyme site for confirmation. The repair DNA was amplified by PCR and was cotransformed with the plasmid that contains the sgRNA and Cas9. To inactivate *cek1* and *cek2*, stop codons were introduced in each gene (19th and 25th amino acid codons in *cek1* and 32nd and 37th amino acid codons in *cek2*). The screening primers were designed to amplify around 1 kb for sequencing. All the oligonucleotides used in this experiment are listed in Table S1.

### Mating assays.

Opaque colonies were identified on SC-GlcNAc agar medium with 5 μg/ml Phloxine B after 5 days of growth at room temperature (RT). Opaque cells of strain 3315 α/α (−Trp, −Lys) were used as the tester strain for mating. Opaque colonies of *MTL***a/a** versions of CAI4, Cpp1Δ/Δ, Cek1Δ/Δ, GR001, GR002, GR003, and GR004 strains (all −Ura) were restreaked on separate YPD, SC-GlcNAc, and SC-glucose agar plates from the experimental strains. Opaque cells of tester strains were streaked on YPD plates. After 48 h of incubation at RT, the two sets of tester and experimental streaks were replica plated together onto both SC-GlcNAc and SC-glucose plates. After 48 h on SC-GlcNAc and SC-glucose plates at RT, cells were replicated onto SC-glucose selection medium lacking uridine, tryptophan, and lysine for prototrophic selection. After 3 days of incubation at 30°C, all plates were scanned and replica plated again on the selection medium (−Trp −Lys −Ura) for further confirmation of stable prototrophic colonies (29).

### Quantitative mating assays.

Quantitative mating assays were done in liquid SC-glucose medium. Opaque cells of strain 3315*α/α* were used as the tester strains for mating, and CAI4, Cpp1Δ/Δ, Cek1Δ/Δ, GR001, GR002, GR003 and GR004 strains, all *MTL***a/a,** were tested as experimental strains. Opaque cells were selected from SC-glucose agar medium containing Phloxine B (5 μg/ml) after 5 days of culture at RT. Cells were cultured separately in liquid SC-glucose medium at RT with shaking at 220 rpm for 24 h. Equal amounts of cells (1 × 10^7^) from each strain were adjusted after quantification with a hemocytometer at ×400 magnification. After centrifuging the cells, tester and experimental strains were mixed in separate 5-ml amounts of fresh SC-glucose liquid medium in 50-ml tubes. Cells were gently (220 rpm) shaken for 48 h at RT. Cells were then plated onto selection plates for 3 days at 25°C before counting the colonies (30).

To calculate the mating frequency, the number of prototrophic mating product colonies on the Trp^−^ Lys^−^ Ura^−^ plates was divided by the input on Ura^−^ plates. The experiments were done twice, and the average value is shown (Table 4).

### Pheromone response assays.

Opaque cells of all strains were incubated in SC-glucose liquid medium for 24 h at RT. Cells were diluted for a final OD_600_ of 0.8 and treated with C. albicans α pheromone (1 µg/ml) (Sigma) for 3 h, 6 h, and 24 h before being photographed and scored for projection formation. Strains were visualized using a Nikon Eclipse TS100 microscope with ×400 magnification using differential interference contrast (DIC) optics. A full list of gene expression values after pheromone treatments of the WT and mutant strains is given in an Excel file at GEO under accession number GSE125636.

### Halo assays.

For halo assays, 5 µl of C. albicans α-factor (Sigma) dissolved in 50% methanol (1 µg/ml) was spotted directly onto a lawn of freshly seeded opaque cells. Opaque cells were identified on SC-glucose medium containing Phloxine B (5 μg/ml). A single colony was diluted into 800 µl of sterile water, and serial dilutions were done (1/2 and 1/4) to identify the optimal lawn concentration of cells; 150 µl of each dilution was spread onto separate SC-glucose plates, 5 µl α-pheromone was added, and cells were grown for 24 h at RT. Pictures of plates were scanned at 300 dpi using an Epson 3700 dpi scanner.

### RNA sequencing.

Total RNA was extracted from CAI4, Cpp1Δ/Δ, Cek1Δ/Δ, GR001, GR002, GR003 and GR004 strains. Cultures were grown overnight in SC-glucose medium at RT, diluted to an OD_600_ of 0.1 in 10 ml SC-glucose medium, and cultured to reach an OD_600_ of 0.8 to 1 the next day. For each strain 2 sets of samples were prepared; one set was treated with α-pheromone (1 μg/ml) and the other was untreated. After 3 h of treatment, cells were collected at an OD_600_ of between 0.8 and 1. Total RNA was extracted according to the Qiagen RNeasy minikit protocol, and the cells were disrupted with bead beater shaking 25 times for 20 s with 1 min of cooling on ice between treatments. RNA quality and quantity were determined using an Agilent 2100 Bioanalyzer (Agilent Technologies, USA). Library preparation and RNA sequencing were carried out at the Quebec Genome Innovation Center located at McGill University using an Illumina MiSeq sequencing platform. RNA-seq data were processed as described previously (31).

### Phosphorylation analysis and Western blotting.

White and opaque cells of all strains were grown in SC-glucose liquid medium for 24 h at RT. Cells were then diluted to an OD_600_ of 1.0 at 25°C in both the presence and absence of pheromone. After 3 h, cells were harvested by centrifugation, washed with IP150 buffer (50 mM Tris-HCl [pH 7.4], 150 mM NaCl, 2 mM MgCl_2_, 0.1%NP-40), and lysed with glass beads in IP150 buffer supplemented with a protease inhibitor cocktail tablet (Roche), anti-phosphatase inhibitor (Roche), and 1 mM phenylmethylsulfonyl fluoride (PMSF). The protein extract was clarified by centrifugation for 1 min at 13,000 rpm at 4°C, the protein concentration was measured by Bradford’s method, and equal amounts of proteins were boiled with SDS gel loading buffer and resolved in 4 to 20% gradient SDS polyacrylamide gels. The separated polypeptides were transferred electrophoretically onto a nitrocellulose membrane and analyzed using anti-P44/42 MAPK polyclonal antibody (Cell Signaling catalog no. 9101). The membrane was developed using the Li-Cor Odyssey system using their anti-rabbit-IR-Dye 680-conjugated secondary antibody (32).

### Cek1 tagging with GFP.

The GFP along with the marker *URA3* was amplified by PCR from the plasmid pFA-GFP-CaURA3 using primers that provided 80-nt homology to regions before the stop codon of *CEK1* and to flanking regions downstream of *CEK1* for the construction of Cek1-GFP by using standard methods. The PCR product was used to transform the CAI4 and Cpp1Δ/Δ parental strains using the lithium acetate method of transformation (33), and the transformants were selected from SC-Ura^−^ agar plates. Successful transformants of Cek1-GFP were confirmed by colony PCR.

### Cek2 tagging with GFP.

The C. albicans CAI4 and Cpp1Δ/Δ strains were transformed with a PCR product (designed sgRNA and repair DNA) to C-terminally tag the gene *CEK2* (*ORF19.460*) with green fluorescent protein (GFP) using the transient CRISPR-Cas9 system described by Min et al. (34), using the “solo system” strategy for C. albicans (27). Three sets of primers were designed for the CRISPR mutant construction: sgRNA, repair DNA, and screening primers. To design the sgRNA, Benchling (Biology Software, 2018) was used based on the following guidelines: single guide; guide length, 20; genome, CA22; PAM, NGG. A guide sequence was chosen near the stop codon with higher on-target and lower off-target scores. The GFP with the *URA3* selection marker was amplified by PCR from the plasmid pFA-GFP-CaURA3 before the stop codon of *CEK2* and to flanking regions downstream of *CEK2*. The repair DNA was amplified by PCR and was cotransformed with the plasmid that contains the sgRNA and Cas9 (33). All the oligonucleotides used in this experiment are listed in Table S1.

### Immunofluorescence microscopy.

Cells from single colonies were grown in 10 ml SC-glucose liquid medium for 24 h at RT, with shaking at 220 rpm. Cells were diluted to a final OD_600_ of 0.8, and each sample was divided into two; half was treated with α-pheromone (1 µg/ml) for 3 h and the other half was untreated. After 3 h, 100 µl of the cultures was washed with MilliQ water and was stained with 2 µl/ml of DAPI for 5 min before washing in water and preparing slides for Leica DM600 microscopy.

### Data availability.

Original data files are available at GEO under accession number GSE125636.
