# Supplementary material for: MAP Kinase Regulation of the Candida albicans Pheromone Pathway
Source: mSphere. 2019 Feb 20;4(1):e00598-18. doi: 10.1128/mSphere.00598-18 (PMC6382970; doi:10.1128/mSphere.00598-18)
Supplement: TABLE S1 [file mSphere.00598-18-st001.docx]

Table S1

| **Name** | **Description** | **Sequence (5' to 3')** | **Source** |
| --- | --- | --- | --- |
| ***CEK2* deletion** | **SgRNA Forward** | atttgTGCTTATGGCATTGTTGCTTg | This study |
|  | **SgRNA Reverse** | aaaacAAGCAACAATGCCATAAGCAc | This study |
|  | **Repair DNA Forward** | atatctcaaaactttaaagtgctcaagattttgggagagggtgcttaAggAattCttgcttAgC | This study |
|  | **Repair DNA Reverse** | tttttttgatggccaccttagtctctgtgggtaaatgtactgGcTaagcaaGaatTccTtaagc | This study |
|  | **Checking primers Forward** | tacaaccaagttttacaatacctg | This study |
|  | **Checking primers Reverse** | agataaacttcattgaagctgtc | This study |
| ***CEK1* deletion** | **SgRNA Forward** | Atttgacaacaacaagctcaggctcg | This study |
|  | **SgRNA Reverse** | aaaacgagcctgagcttgttgttgtc | This study |
|  | **Repair DNA Forward** | caa cat cat cag ctt caa cag caa cat caa caa caa atg tt aTaacaacGaATtcaggct Tag gc | This study |
|  | **Repair DNA Reverse** | gttgttgttgttgttgttgttgtgcttgagcttgagcctgagcctAagcctgaATtCgttgttAt | This study |
|  | **Checking primers Forward** | tgaatatttcgaccacgtca | This study |
|  | **Checking primers Reverse** | agctacgtattctgtcatga | This study |
| ***Cek1*-GFP** | **CEK1-S1-GFP** | TCGATTTTGATAAAATGAAAGATCAATTAACAATTGAAGATTTGAAAAAATTGTTATATGAAGAGATTATGAAGCCATTA ggtgctggcgcaggtgcttc | This study |
|  | **CEK1-S2-GFP** | CCTATACAACAACAATTATGCTAAATCTACAACAACTACCAAGCCCAACCTATAGTTTTTAGTTTAGTTTAGTTTAGTTT tctgatatcatcgatgaattcgag | This study |
|  | **CEK1-CHK-F** | TACCAACAACAATACTAGTAC | This study |
|  | **CEK1-CHK-R** | CGGTGTTATTAAATCTCCTA | This study |
| ***Cek2-GFP*** | ***SgRNA Forward*** | atttgattatactttataaagatatg | This study |
|  | ***SgRNA Reverse*** | aaaacatatctttataaagtataatc | This study |
|  | ***Repair DNA Forward*** | aagagtttgattttgatatagataagaagaatttggacaccaatgacttgaaaaaacaaattttcgaaatagtCATGTCG **GGTGCTGGC**GCAGGTGCTTC | This study |
|  | ***Repair DNA Reverse*** | acagggttgaacagtgaacccaataaaagttttaacagatattattgaaagacaaagaaaacatcactaaacaaattaaaTTATTTGTATAGTTCATCCA | This study |
|  | ***Checking primers Forward*** | cttgatgagcctgttacact | This study |
|  | ***Checking primers Reverse*** | gagaaaagatgatcgctatc | This study |
